# Prevalence of Diabetic Retinopathy in Mainland China: A Meta-Analysis

**DOI:** 10.1371/journal.pone.0045264

**Published:** 2012-09-20

**Authors:** Lei Liu, Xiaomei Wu, Limin Liu, Jin Geng, Zhe Yuan, Zhongyan Shan, Lei Chen

**Affiliations:** 1 Department of Ophthalmology, The First Hospital of China Medical University, Shenyang, People's Republic of China; 2 Department of Clinical Epidemiology and Evidence Medicine, The First Hospital of China Medical University, Shenyang, People's Republic of China; 3 Key Laboratory of Endocrine diseases in Liaoning Province, The First Hospital of China Medical University, Shenyang, People's Republic of China; 4 Liaoning Diabetic Eye Center, The First Hospital of China Medical University, Shenyang, People's Republic of China; Massachusetts Eye & Ear Infirmary, Harvard Medical School, United States of America

## Abstract

**Background:**

Although diabetic retinopathy (DR) is considered to be a major cause of blindness, this is the first meta-analysis to investigate the pooled prevalence of DR in mainland China.

**Methodology/Principal Findings:**

We conducted a search of all English reports on population-based studies for the prevalence of DR using Medline, EMbase, Web of Science, Google (scholar), and all Chinese reports were identified manually and on-line using CBMDisc, Chongqing VIP database, and CNKI database. A meta-analysis was carried out. The fixed effects model or random effects model was used as a statistical test for homogeneity. Nineteen studies were included. The prevalence of DR, non-proliferative diabetic retinopathy (NPDR) and proliferative diabetic retinopathy (PDR) in the pooled general population was 1.3% (95%CI: 0.5%–3.2%), 1.1% (95%CI: 0.6%–2.1%), and 0.1% (95%CI: 0.1%–0.3%), respectively, but was 23% (95%CI: 17.8%–29.2%), 19.1% (95%CI: 13.6%–26.3%), and 2.8% (95%CI: 1.9%–4.2%) in the diabetic group. The prevalence rate of DR in the pooled rural population was higher than that in the urban population, 1.6% (95%CI: 1.3%–2%), and the diabetic population, 29.1% (95%CI: 20.9%–38.9%). The prevalence of DR was higher in the Northern region compared with the Southern region.

**Conclusions/Significance:**

The prevalence of DR in mainland China appeared a little high, and varied according to area. NPDR was more common. This study highlights the necessity for DR screening in the rural areas of China.

## Introduction

Diabetic retinopathy (DR) is one of the foremost causes of blindness in the working age population [1]. As the prevalence of diabetes mellitus (DM) increases globally and patients live longer, the development of DR as a microvascular complication of DM also rises. DR is a priority disease in the “VISION 2020” initiative for the global elimination of avoidable blindness. The World Health Organization (WHO) has recommended its member countries integrate a program approach for DR within their prevention of blindness programs. In contrast to studies in Western countries, age-related macular degeneration (AMD) and DR appear to play a minor role as a cause of visual impairment in elderly Chinese [2].

In mainland China, many previous reports have shown the prevalence of DR in population-based studies, however, the prevalence of DR varies in different samples, and the data remain limited and localized. To date, there is no national, population-based study of the prevalence of DR in mainland China, and it would seem that a national, pooled estimate based on the mainland population is necessary. Satisfactory and reasonable prevalence estimates will be useful for China to manage this problem. In this meta-analysis, we carried out a systematic review of previous population-based studies on DR prevalence in mainland China, and investigated any differences among areas. The results are presented here.

## Methods

### Search Strategy

We searched all English reports on population-based studies for the prevalence of DR using Medline, EMbase, Web of Science, Google (scholar), and all Chinese reports were searched manually and on-line using CBMDisc (Chinese Biochemical Literature on Disc), Chongqing VIP database, and CNKI (China National Knowledge Infrastructure) database. The search keywords were: diabetic retinopathy, prevalence, and Chinese population. A total of 196 reports published in the period from 1991 to 2012 were identified.

### Inclusion and Exclusion Criteria

**Figure 1:**
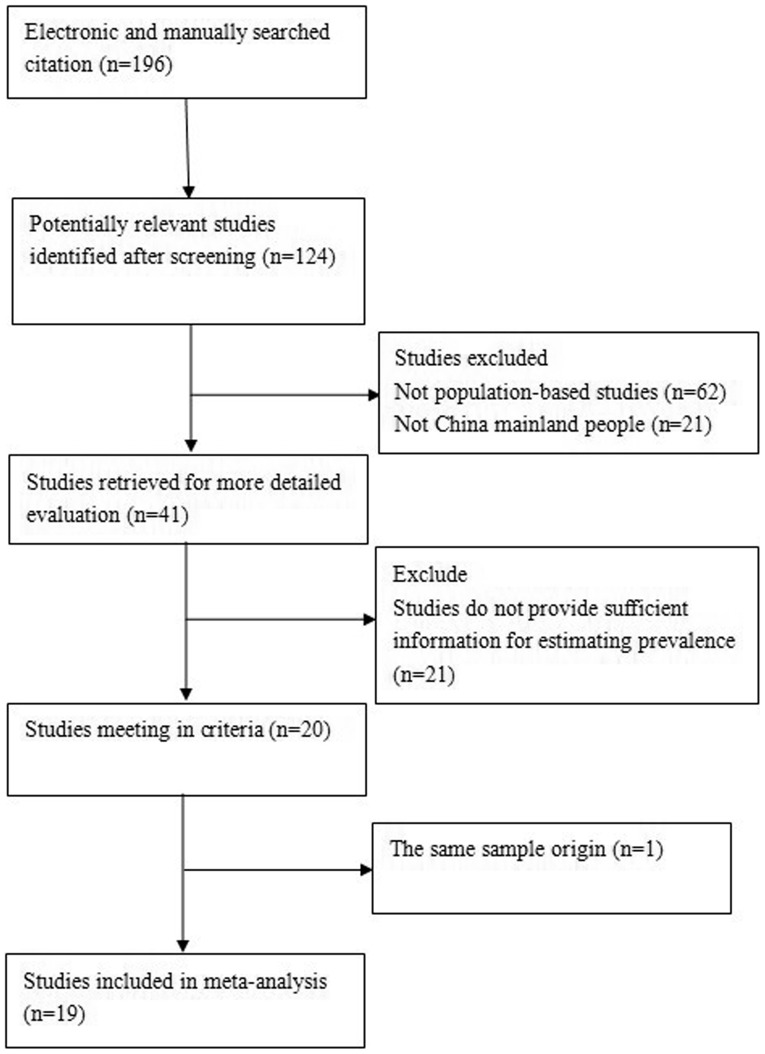
Flow chart demonstrating those studies that were processed for inclusion in the meta-analysis.

Reports potentially eligible for inclusion in this meta-analysis had to meet the following criteria: they had to be population-based studies, subjects had to be in mainland China, and the studies needed to provide sufficient information to estimate the pooled prevalence of DR. If more than one study was based on the same population sample, the study of the highest quality was included. Reports were excluded for the following reasons: the study was on a duplicate population group but of lower quality, and the study did not satisfy one or more inclusion criteria. A total of 196 potentially relevant studies were identified and screened. After systematic review, only 19 of these were included in the meta-analysis. The progress for study inclusion is shown in Figure 1.

### Data Extraction

The literature was searched independently by two researchers (Lei Liu and Jin Geng). Data were extracted from each article using a standardized form, designed in advance. The characteristics of the population-based studies included in this meta-analysis on the pooled prevalence of DR in mainland China are shown in Table 1.

**Table 1:** Characteristics of population-based studies on the prevalence of diabetic retinopathy (DR) in mainland China.

First Author	Publication year	Area	Region	Rural/Urban	Examination methodolo gies for DR	Diagnosis standard for DR	Diagnosis standard for DM	Age (year)	Survey day	Sample (n)	DM (n)	DR (n)		
												total	NPDR	PDR
Yinghua HU [17]	1991	Daqing	Northern	Urban	MDOC+fundus photography	NA	WHO	25–74	1986	110660	423	129	119	10
Shouzhi He [18]	1997	Beijing	Northern	Urban	MDOC+FFA	Staging of DR for Chinese*	WHO	>30	NA	29938	534	90	88	2
Shouling Li [19]	1998	Anhui	Southern	Rural+Urban	MDOC	Staging of DR for Chinese*	WHO	>15	1994–1995	11618	216	67	NA	NA
Guanglu Wang [20]	2001	Beijing	Northern	Rural+Urban	MDOC	NA	WHO	≥25	1994–1995	1483	326	37	30	7
Haidong Zhou [21]	2006	Shanghai	Southern	Urban	NMFP for 45 digree field	Staging of DR for Chinese*	NA	44–87	Jul 2003–Apr 2004	43762	535	146	123	23
Xianjun Liang [22]	2006	Foshan	Southern	Urban	MDOC	NA	WHO	>18	2003–2005	10723	356	38	NA	NA
Xiwei Xie [23]	2009	Beijing	Northern	Rural+Urban	MFP for 50 digree field	ETDRS	Self-reported history	>40	2001	4391	434	285	273	12
Lei Liu [24]	2009	Shenyang	Northern	Urban	NMFP for 45 digree field	ETDRS	WHO	14–82	Oct-Dec 2007	1534	137	17	17	NA
Xiangwen Shu [25]	2010	Shandong	Northern	Rural	MDOC	ETDRS	WHO	>25	2007–2008	16330	689	181	107	74
Zhong Xin [26]	2010	Beijing	Northern	Rural	MFP for seven field	Proposed international severity scales**	WHO	>35	May-Jul 2008	1293	114	27	25	2
Yan Teng [27]	2010	Shuangcheng	Northern	Rural	MFP for 45 digree field	Staging of DR for Chinese*	WHO	>50	Nov 2006–Feb 2007	5053	NA	56	49	7
Hailian Dong [28]	2010	Shunyi	Northern	Rural	NMFP for three field		NA	>18	Jan-Dec 2009	5734	554	94	76	18
Mingzhu Ynag [29]	2010	Shijiazhuang	Northern	Urban	MDOC	Staging of DR for Chinese*	WHO	35–80	Sep 2006-Sep 2008	3318	381	87	69	18
Hongbo Wang [30]	2010	Changzhi	Northern	Rural	NMFP for 45 digree field	Staging of DR for Chinese*	WHO	>15	Oct 2007-Dec 2008	57500	2632	986	NA	NA
Xiuqun Ye [31]	2010	Huizhou	Southern	Urban	MDOC+fundus photography+FFA	ETDRS	WHO	>20	2003–2005	11723	1046	101	71	30
Fenghua Wang [32]	2011	Handan	Northern	Rural	MFP for two field	ETDRS	self-reported history and FPG	>30	2006–2007	6830	368	165	145	20
Bingzhen Li [33]	2011	Beijing Shunyi	Northern	Urban	NMFP for one field	ETDRS	WHO	≥40	May-Oct 2005	4167	445	130	124	6
Can Pang [34]	2011	Shanghai	Southern	Urban	NMFP for 45 digree field	Diabetic Retinopathy Disease Severity Scale	OGTT	>15	1996–2007	3259	799	75	74	1
Jie Xu [35]	2012	Beijing	Northern	Urban	NMFP for 45 digree field	ETDRS	WHO	20–80	August 2008-July 2009	NA	2007	496	329	67

Abbreviation: ETDRS, Early Treatment Diabetic Retinopathy Study; DR, diabetic retinopathy; NPDR, non-proliferative diabetic retinopathy; PDR, proliferative diabetic retinopathy. DM, diabetes mellitus; MDOC, mydriasis direct ophthalmoscope check; FFA, fundus fluorescein angiography; NMFP, non-mydriatic fundus photography; MFP, mydriatic fundus photography. NA, not available.

* Staging of DR by Chinese academy of ocular fundus disease.

** Proposed international clinical diabetic retinopathy and diabetic macular edema disease severity scales.

### Data Analysis

Meta-analyst statistical software offered by http://tuftscaes.org/meta_analyst/was used to analyze the data. The pooled prevalence was used to estimate the prevalence of DR in China mainland. All meta-analyses were evaluated for heterogeneity using the Chi-square based Q test and *I* ^2^ test [3]. *I* ^2^ estimated the percentage of the total variance in all of the data under consideration that was related to heterogeneity. The authors suggested using 25%, 50%, and 75% to indicate low, moderate, or high level heterogeneity. If there was moderate or high level heterogeneity, a random-effects meta-analysis was performed by the DerSimonian and Laird method, unless using fixed-effects models. Publication bias was assessed by visually inspecting a funnel plot. A p value less than 0.05 was considered statistically significant [4], [5]. The results were analyzed statistically using the χ^2^ (Chi-square) test with the SPSS 13.0 program (SPSS Software, Chicago, USA) to compare the difference between two groups with different prevalence rates.

**Figure 2:**
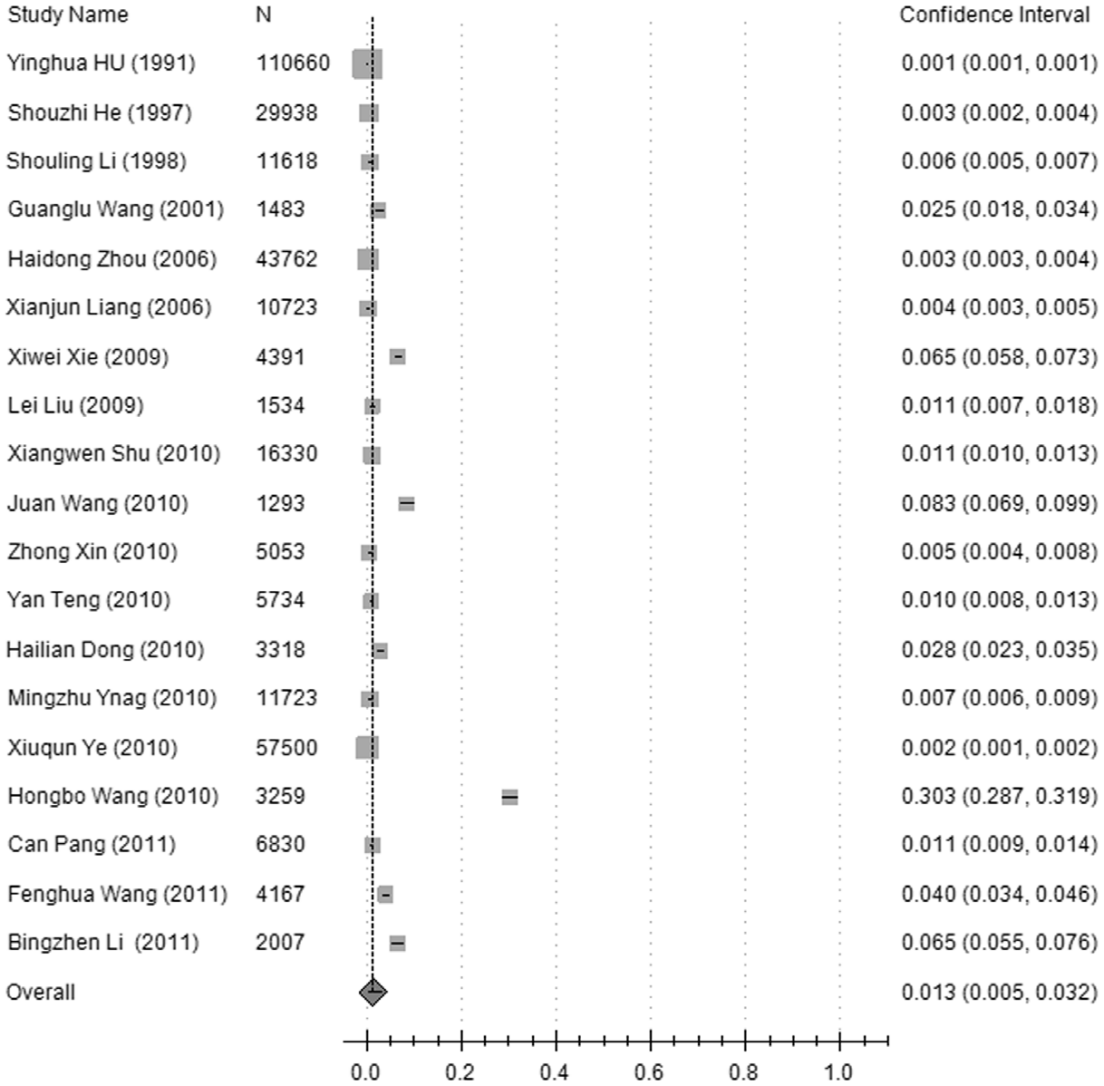
A forest plot displaying the pooled prevalence of diabetic retinopathy (DR) in the population of mainland China.

## Results

**Figure 3:**
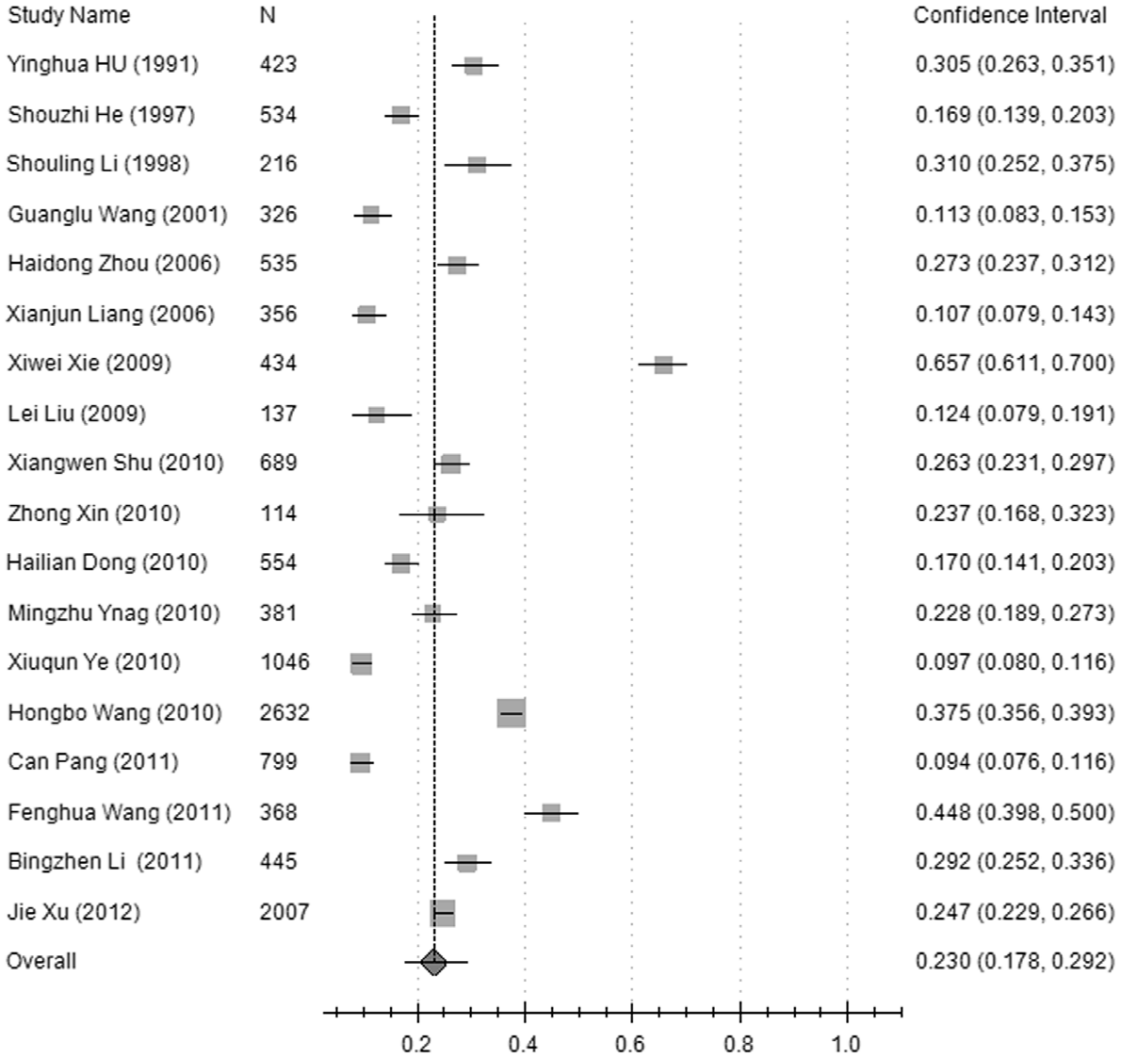
A forest plot displaying the pooled prevalence of diabetic retinopathy (DR) in the diabetic population of mainland China.

**Figure 4:**
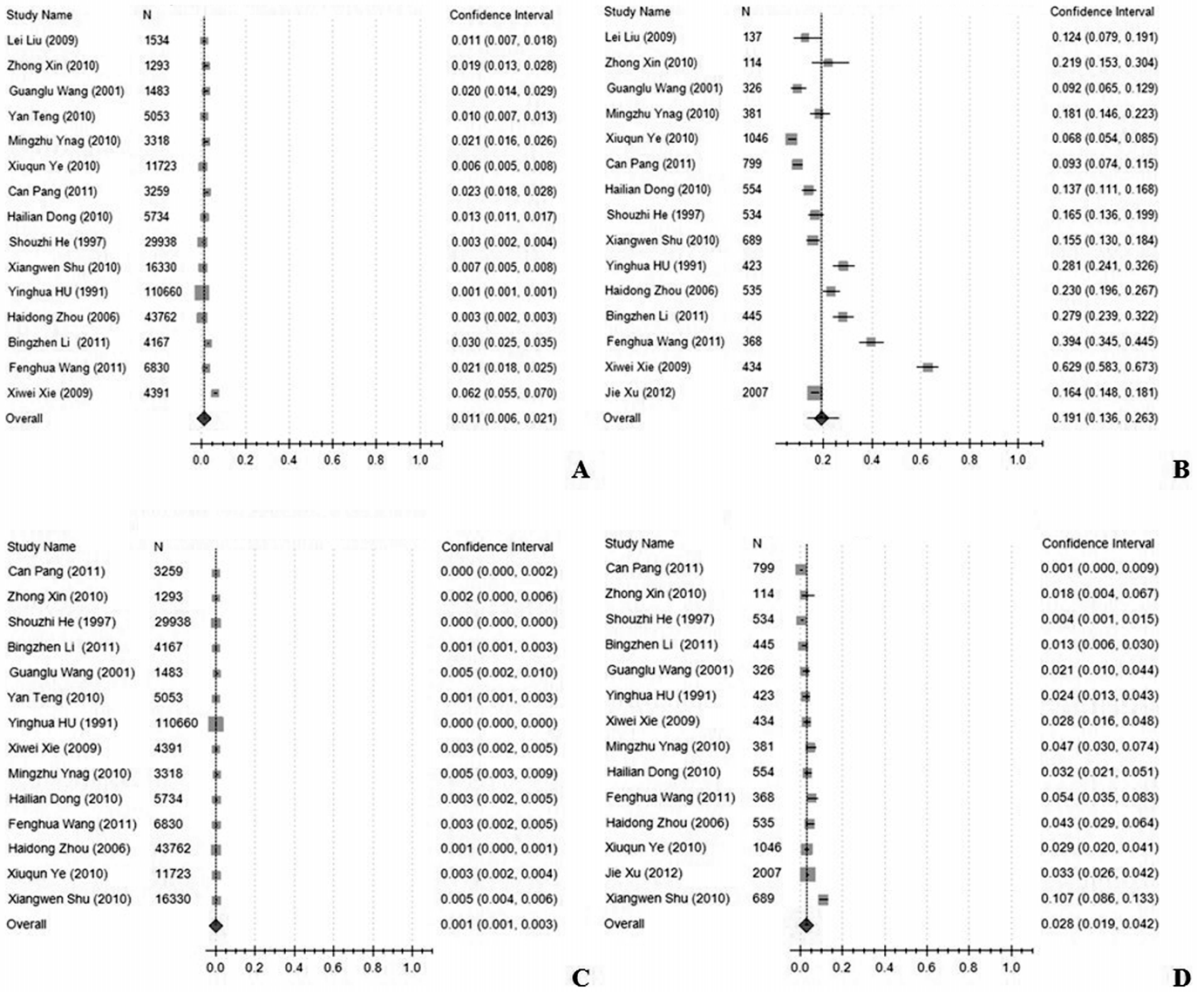
A forest plot displaying the pooled prevalence rates of NPDR and PDR (A, NPDR in general mainland China population; B, NPDR in diabetic population; C, PDR in general mainland China population; D, PDR in diabetic population).

The prevalence of DR, NPDR and PDR in the pooled general population was 1.3% (95%CI: 0.5%–3.2%), 1.1% (95%CI: 0.6%–2.1%), and 0.1% (95%CI: 0.1%–0.3%), respectively, but was 23% (95%CI: 17.8%–29.2%), 19.1% (95%CI: 13.6%–26.3%), and 2.8% (95%CI: 1.9%–4.2%) in the diabetic group. The pooled prevalence rates of DR were higher in the rural population, 1.6% (95%CI: 1.3%–2%), compared with the urban population, 0.8% (95%CI: 0.3%–1.7%), (χ^2^ = 771.35, *p*<0.001), and higher in the rural diabetic group, 29.1% (95%CI: 20.9%–38.9%) compared with the urban diabetic group, 18.1% (95%CI: 13.6%–23.7%) (χ^2^ = 263.37, *p*<0.001),. In the general population, the pooled prevalence of DR was higher in the Northern region of China, 1.4% (95%CI: 0.8%–2.5%), compared with the Southern region, 0.7% (95%CI: 0.3%–1.4%), (χ^2^ = 115.91, *p*<0.001). In the diabetic group, the pooled prevalence of DR in the Northern region of China, 26.5% (95%CI: 20.6%–33.3%), was also higher compared with the Southern region, 15.7% (95%CI: 8.9%–26.3%) (χ^2^ = 281.61, *p*<0.001). The data are shown in Figure 2, 3, and 4, and Table 2.

**Table 2:** The pooled prevalence of diabetic retinopathy (DR) in mainland China with comparisons between different regions (North vs. South and rural vs. urban).

	Region	No.of articles	Case/Total	Pooled Estimate (%)	95%CI(%)	HeterogeneityI^2^ (%)	Q value	*p* value
General population	Urban	9	1309/219084	0.8	0.3–1.7	49.80	0.99	<0.001
	Rural	6	1509/92740	1.6	1.3–2	48.00	0.98	<0.001
Diabetics	Urban	10	1309/6663	18.1	13.6–23.7	49.00	0.99	<0.001
	Rural	5	1453/4357	29.1	20.9–38.9	49.20	0.99	<0.001
General population	Northern	13	2284/248231	1.4	0.8–2.5	49.80	0.99	<0.001
	Southern	5	427/81085	0.7	0.3–1.4	49.50	0.99	<0.001
Diabetics	Northern	13	2724/9044	26.5	20.6–33.3	49.40	0.99	<0.001
	Southern	5	427/2952	15.7	8.9–26.3	49.30	0.99	<0.001

**Figure 5:**
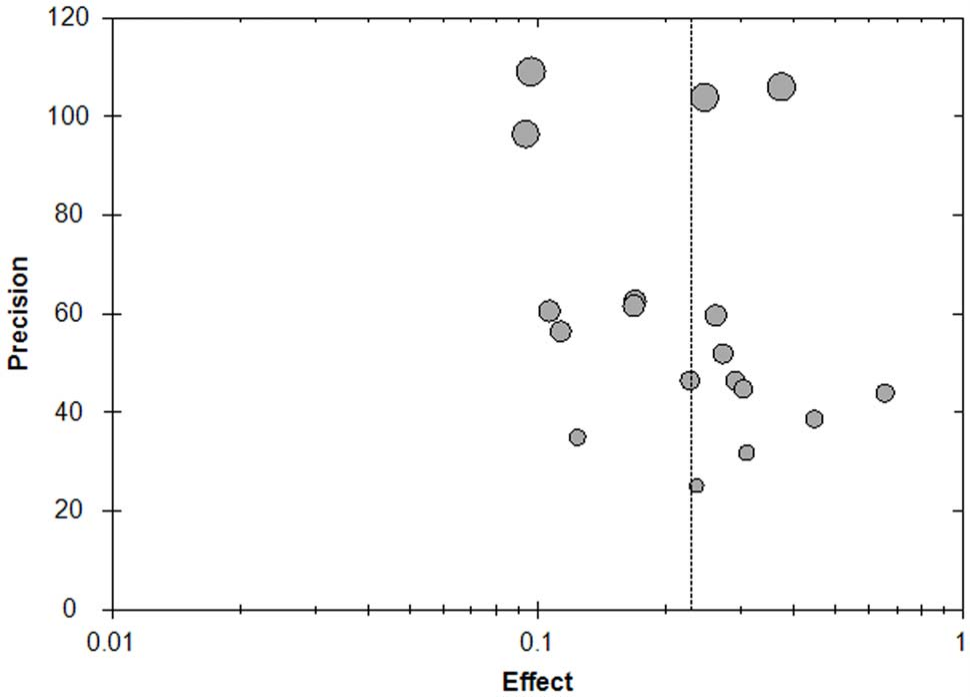
A funnel plot of studies conducted on the prevalence of diabetic retinopathy (DR) in mainland China.

All comparisons passed the test of heterogeneity, as previously defined Random-effects models were used for meta-analyses. There was no significant publication bias in this meta-analysis. The funnel plot of prevalence of DR in diabetes is shown in Figure 5.

## Discussion

To our knowledge, this is the first meta-analysis of DR prevalence in mainland China. In this meta-analysis, a total of 19 studies with 329,316 samples were included. We showed that the pooled prevalence rate of DR was 1.3% in the general population, and 23% (95%CI: 17.8%–29.2%) in the diabetic group. In mainland China there are 31 provinces (including 22 provinces, four municipalities, five autonomous regions) totally. The eligible studies covered 12/31 Chinese provinces. China mainland's population has increased to 1.3 billion, including 92.4 million diabetics [6], inferring (from our results) 16.9 million with DR, and 21.2 million diabetics with DR, respectively. This indicates a strong requirement for prevention and treatment strategies to control DR.

A previous study suggested that the overall, age-standardized prevalence of DR was 20.1% in Chinese diabetics living in Singapore [7]. The prevalence was lower than that of this meta-analysis (23%). However, in a US population of Chinese people with diabetes, the prevalence of DR was even higher (25.7%) [8]. In Taiwan, the prevalence of DR in diabetes was shown to be 35% [9], but 18.2% in Hong Kong [10]. Thus, the prevalence of DR in Chinese diabetics living abroad appeared to be a little lower than in mainland China, except for those living in the U.S. and Taiwan.

The pooled prevalence rate of DR in the rural population was higher than that in the urban population. This was similar to a study conducted in India [11]. The reasons for this discrepancy in a developing country may be: a lower socioeconomic life, a poorer health care system, and fewer social activities for Chinese rural residents [12].

The pooled prevalence of DR was higher in the Northern region compared with the Southern region, both for the general population (1.4% compared with 0.7%), and the diabetic population (26.5% compared with 15.7%). A different life style and socioeconomic status may play an important role in the discrepancy of the pooled prevalence of DR between the Northern and Southern regions. The per capita Gross Domestic Product (GDP) is a little higher in Northern than that in Southern regions (http://tieba.baidu.com/f?kz=1064355963). In addition, urbanization is associated with changes in a number of lifestyle factors, such as physical inactivity, unhealthy diet, and obesity, which are implicated in the aetiology of diabetes especially in Northern of China. As the apparent epidemic of diabetes occurrence with socioeconomic and lifestyle changes, diabetic complications, such as DR are also prevalent in developed societies. Previous studies also showed us that the prevalence of diabetes was higher in residents of Northern compared to Southern in China. As the prevalence of DM rising, we can conclude the prevalence of DR is a little higher in population or diabetic population of Northern compared to Southern in mainland China [13]–[16]. In addition, a difference in the number of studies conducted in the two regions may also explain this discrepancy. Thirteen of the studies on DR prevalence were conducted in the Northern region, but only five were from the Southern region.

As shown in the results (Table 2), there were moderate heterogeneity among included studies. In this meta-analysis, the different prevalence rates of DR might have also impacted by the heterogeneity of studies with different examination methodologies and ophthalmologic definitions. For example, between populations of different regions, the prevalence rates of DR were reported 26.27% in Shandong rural diabetes but 16.97% in Beijing Shunyi county rural diabetes. Similarly, between different diabetes of the same region the prevalence rates of DR were varied from 24.71% in Beijing Shunyi to 16.85% in other Beijing samples. According to the different population characteristics and data pooled from studies at different time points, the prevalence rates of DR might also be varied. However, the pooled prevalence rates of DR were also estimated by Random-effects meta-analysis model.

Although we have estimated the pooled prevalence of DR in mainland China, which is very important for preventative public health, there were some limitations in this meta analysis. First, we used pooled prevalence data using meta-analysis, rather than the prevalence in a single national population-based study. Second, as we cannot have access to unpublished results, a publication bias cannot be excluded. Third, there were limited studies in the Southern areas in China.

In view of the results of this meta-analysis, and given that the prevalence rates of DR appeared to vary in different regions and population groups, it would seem that we need to prioritize the treatment of DR in the rural population.

## References

[1] Yamada M, Hiratsuka Y, Roberts CB, Pezzullo ML, Yates K, et al (2010) Prevalence of visual impairment in the adult Japanese population by cause and severity and future projections. Ophthalmic Epidemiol 17: 50–57. 20100100 10.3109/09286580903450346

[2] Xu L, Wang YX, Li YB, Wang Y, Cui TT, et al (2006) Causes of blindness and visual impairment in urban and rural areas in Beijing: the Beijing Eye Study. Ophthalmology. 113: 1134–1141. 10.1016/j.ophtha.2006.01.035 16647133

[3] Higgins JP, Thompson SG, Deeks JJ, Altman DG (2003) Measuring inconsistency in meta-analyses. BMJ 327: 557–560. 12958120 10.1136/bmj.327.7414.557 PMC192859

[4] Mantel J, Haenszel W (1959) Statistical aspects of the analysis of data from retrospective studies of disease. J Natl Cancer Inst 22: 719–748. 13655060

[5] Der Simonian R, Laird N (1986) Meta-analysis in clinical trials. Control Clin Trials 7: 177–188. 3802833 10.1016/0197-2456(86)90046-2

[6] Yang W, Lu J, Weng J, Jia W, Ji L, et al (2010) Prevalence of diabetes among men and women in China. N Engl J Med 362: 1090–1101. 20335585 10.1056/NEJMoa0908292

[7] Chiang PP, Lamoureux EL, Cheung CY, Sabanayagam C, Wong W, et al (2011) Racial differences in the prevalence of diabetes but not diabetic retinopathy in a multi-ethnic Asian population. Invest Ophthalmol Vis Sci 52: 7586–7492. 21862647 10.1167/iovs.11-7698

[8] Wong TY, Klein R, Islam FM, Cotch MF, Folsom AR, et al (2006) Diabetic retinopathy in a multi-ethnic cohort in the United States. Am J Ophthalmol 141: 446–455. 16490489 10.1016/j.ajo.2005.08.063 PMC2246042

[9] Chang C, Lu F, Yang YC, Wu JS, Wu TJ, et al (2000) Epidemiologic study of type 2 diabetes in Taiwan. Diabetes Res Clin Pract 50 Suppl 2 S49–59. 11024584 10.1016/s0168-8227(00)00179-0

[10] Lee KM, Sum WM (2011) Prevalence of diabetic retinopathy in patients with recently diagnosed diabetes mellitus. Clin Exp Optom 94: 371–375. 21323731 10.1111/j.1444-0938.2010.00574.x

[11] Rani PK, Raman R, Sharma V, Mahuli SV, Tarigopala A, et al (2007) Analysis of a comprehensive diabetic retinopathy screening model for rural and urban diabetics in developing countries. Br J Ophthalmol 91: 1425–1429. 17947265 10.1136/bjo.2007.120659 PMC2095459

[12] Zhang L, Xu Y, Nie H, Zhang Y, Wu Y (2012) The prevalence of depressive symptoms among the older in China: a meta-analysis. Int J Geriatr Psychiatry doi: 10.1002/gps.2821. 10.1002/gps.2821 22252938

[13] Gu D, Reynolds K, Duan X, Xin X, Chen J, et al (2003) Prevalence of diabetes and impaired fasting glucose in the Chinese adult population: International Collaborative Study of Cardiovascular Disease in Asia (InterASIA). Diabetologia 46: 1190–1198. 12879248 10.1007/s00125-003-1167-8

[14] Cockram CS (2000) The epidemiology of diabetes mellitus in the Asia-Pacific region. Hong Kong Med J 6: 43–52. 10793402

[15] Reynolds K, Gu D, Whelton PK, Wu X, Duan X, et al (2007) Prevalence and risk factors of overweight and obesity in China. Obesity (Silver Spring) 15: 10–18. 17228026 10.1038/oby.2007.527

[16] Sánchez-Thorin JC (1998) The epidemiology of diabetes mellitus and diabetic retinopathy. Int Ophthalmol Clin 38: 11–18. 9604735

[17] Hu YH, Pan XR, Liu PA, Li GW, Howard BV, et al (1991) Coronary heart disease and diabetic retinopathy in newly diagnosed diabetes in Da Qing, China: the Da Qing IGT and Diabetes Study. Acta Diabetol 28: 169–173. 1777654 10.1007/BF00579721

[18] He S, Guo Y, Li Z (1997) Epidemiologic study of diabetic retinopathy in Capital Steel Company. Zhonghua Yan Ke Za Zhi 33: 381–383. 10451988

[19] Shouling Li, Yanfeng Zhou, Di Chen, Minggong Yang, Meiling Zhu (1998) Epidemiological investigation of diabetic retinopathy risk factors. Chin J Ocul Fundus Dis 14: 119–121.

[20] Wang G, Zhang F, Yuan S, Meng S, Zhu L, et al (2001) A screening survey of diabetic retinopathy and other chronic complications in Beijing district. Ophthalmology In China 10: 180–182.

[21] Zou H, Zhang X, Wang F, Xu X, Wang W, et al (2006) Epidemiological investigation of diabetic retinopathy in Beixinjing blocks, Shanghai. Chin J Ocul Fundus Dis 22: 31–34.

[22] Liang X, Lin J, Huang Z, Li G, Wu X (2006) Prevalence and risk factors of diabetic retinopathy in Foshan City. Guangdong Medical Journal 27: 1552–1553.

[23] Xie X, Xu L, Yang H, Wang S, Jonas JB (2009) Frequency of diabetic retinopathy in the adult population in China: the Beijing Eye Study 2001. Int Ophthalmol 29: 485–493. 19221695 10.1007/s10792-008-9272-9

[24] Liu L, Chen L, Hu Y, Liu L (2009) Analysis risk factors for diabetic retinopathy. Shandong Medical Journal 49: 52–53.

[25] Shu X, Wang Y, Fan C, Sheng Y, Zhang H, et al (2010) Epidemiology study on the prevalence rate and risk factors of diabetic retinopathy in rural residents in Shandong. Chin J Ocul Fundus Dis 26: 113–115.

[26] Zhong X, Yahong MA, Lei Z, Yi L, Jing S, et al (2010) Prevalence and risk factors of diabetic retinopathy in rural population of Beijing. Clinical Focus 25: 672–675.

[27] Teng Y, Cui H, Zhang QS, Teng YF, Su Y, et al (2010) Prevalence of diabetic retinopathy among the elderly in rural southern Shuangcheng city, Heilongjiang province. Zhonghua Liu Xing Bing Xue Za Zhi 31: 856–859. 21162981

[28] Dong H (2010) Screening procession for diabetic retinopathy for Shunyi District Houshayu Valley residents. Medical Information 10: 2701–2702.

[29] Yang M, Wei S (2010) Early detection for diabetic retinopathy in Grassroots community and results analysis. Hebei Medical Journal 32: 844–845.

[30] Wang H, Sun F, Zhang Q, Zhai M, Wang S, et al (2010) Epidemiologic study on the prevalence rate and risk factors of diabetic retinopathy in eastern countryside of Changzhi. Chin J Ocul Fundus Dis 26: 109–112.

[31] Ye X (2010) The Analysis about Epidemiological Survey of Diabetic Retinopathy in Huizhou City. Guide of China Medicine 8: 27–28.

[32] Wang FH, Liang YB, Peng XY, Wang JJ, Zhang F, et al (2011) Risk factors for diabetic retinopathy in a rural Chinese population with type 2 diabetes: the Handan Eye Study. Acta Ophthalmol 89: e336–343. 21371287 10.1111/j.1755-3768.2010.02062.x

[33] Li B, Liu Y, Han L, You D, Wang T, et al (2011) Epidemiological survey of diabetic retinopathy in Shunyi district of Beijing. Chin J Exp Ophthalmol 29: 747–752.

[34] Pang C, Jia L, Jiang S, Liu W, Hou X, et al (2012) Determination of diabetic retinopathy prevalence and associated risk factors in Chinese diabetic and pre-diabetic subjects: Shanghai diabetic complications study. Diabetes Metab Res Rev 28: 276–283. 22139892 10.1002/dmrr.1307

[35] Xu J, Wei WB, Yuan MX, Yuan SY, Wan G, et al (2012) Prevalence and risk factors for diabetic retinopathy: the Beijing Communities Diabetes Study 6. Retina 32: 322–329. 21886023 10.1097/IAE.0b013e31821c4252

